# Mass spawning by the date mussel *Lithophaga lithophaga*

**DOI:** 10.1038/s41598-018-28826-8

**Published:** 2018-07-17

**Authors:** Ante Žuljević, Marija Despalatović, Ivan Cvitković, Brian Morton, Boris Antolić

**Affiliations:** 1 Institute of Oceanography and Fisheries, I. Meštrovića 63, 21000 Split, Croatia; 2 School of Biological Sciences, The University of Hong Kong, Hong Kong SAR, China

## Abstract

*Lithophaga lithophaga* is one of the commonest bivalves in the Mediterranean Sea and is present in almost every subtidal calcareous rock. Its reproductive cycle is known only from laboratory studies. Herein, we present data on the species reproductive activities based on localised but mass synchronized spawning events. The species reproduces at the end of the northern hemisphere summer and the majority of significant spawning events occur during the period between full moon and its last quarter. Calm seas are an important pre-requisite for the development of such co-ordinated mass spawning events. ‘Gamete to gamete’ induction seems to be the most likely proximate cue in synchronising gamete release. Spawning begins with a few individuals but spreads progressively along the coastline. In observed situations, reproductive waves finally affect between 10 and >400 m of coastline from 0 to 10 m depth and last longer than three days. In the reproductive zone, dense gamete clouds reduce visibility to zero over tens of metres along the shallow sea bed. No spawning events of such dimensions have been reported upon before for any bivalve.

## Introduction

**Figure 1:**
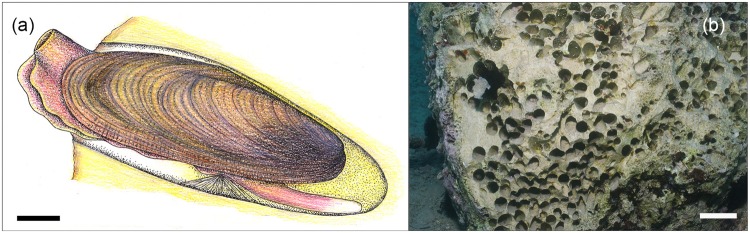
The European date mussel *Lithophaga lithophaga*. (**a**) An illustration of a living individual within its borehole. (**b**) Empty holes after date mussel harvesting indicate population density. Scale bars: (**a**) 1 cm; (**b**) 5 cm.

The European date mussel *Lithophaga lithophaga* (Linnaeus, 1758) (Bivalvia: Mytiloidea: Lithophaginae) is one of the commonest and most well-known bivalves in the Mediterranean Sea and eastern Atlantic^1^. It lives inside subtidal calcareous rocks, which are bored by means of glandular secretions (Fig. 1a)^2,3^. Population densities can be high at >1,500 ind. m^−2^ in shallow waters of the North Adriatic (3–5 m)^4^ or >2,200 in Greece at 6 m depth^5^ while distribution patterns inside rocks are patchy^6^. *Lithophaga lithophaga* grows slowly and a shell length of 5 cm is probably reached between 18–36 years while its life span may be between 54 and 80 years with a maximum shell length of between 8–12 cm^7,8^.

The conservation importance of *L. lithophaga* arises from its gastronomic value and the associated environmental problems resulting from its harvesting. Because the species lives inside rocks, these have to be broken up to expose individuals for collection (Fig. 1b). Such destructive exploitation has affected extensive coastal areas throughout the Mediterranean and Adriatic seas and produced large scale negative impacts on associated benthic communities, which both cover the rock and live inside it^9,10^. It is this destruction that has motivated the protection of the species in the majority of Mediterranean countries. The species is also protected under the *Bern Convention* as a ‘Strictly Protected Faunal Species’, the European *Habitat Directive* as a ‘Fauna and Flora Species of Community Interest that Require Strict Protection’ and the *Barcelona Convention* where it is included in the ‘List of Endangered or Threatened Species’.

The fact that *L. lithophaga* is present in virtually every shallow Mediterranean carbonate rock, both natural and artificially deployed^11^, and reaches high densities, must arise from widescale fertilization success. As with many marine species, the reproductive cycle of *L. lithophaga* is known from laboratory analyses of gonadial development. It is dioecious^7,12,13^ and although hermaphroditism has been observed occasionally this is possibly induced by pollution and endocrine disruption^12^. Sex ratio depends on size but the determined ratio (male:female) varies from 1.13:1 in the Bay of Bizete (Tunisia)^12^, 1.3:1 in Split, Adriatic Sea (Croatia), and 3.1:1 in the Evoikos Gulf (Greece)^7^. In the Mediterranean, *L. lithophaga* becomes reproductively active at an age of ~2^+^ years^7,13^. There is a single reproductive cycle per year and gonad maturation begins in spring and individuals become mature at the end of summer^7,12,13^. Sexual cycle studies have been made on the basis of monthly sampling and have adequately identified the annual reproductive cycle and indicated when spawning probably occurs. Such data, however, have not provided information on spawning synchronicity, if any. Similarly, there are no observations on spawning either in the laboratory or *in situ*.

**Figure 2:**
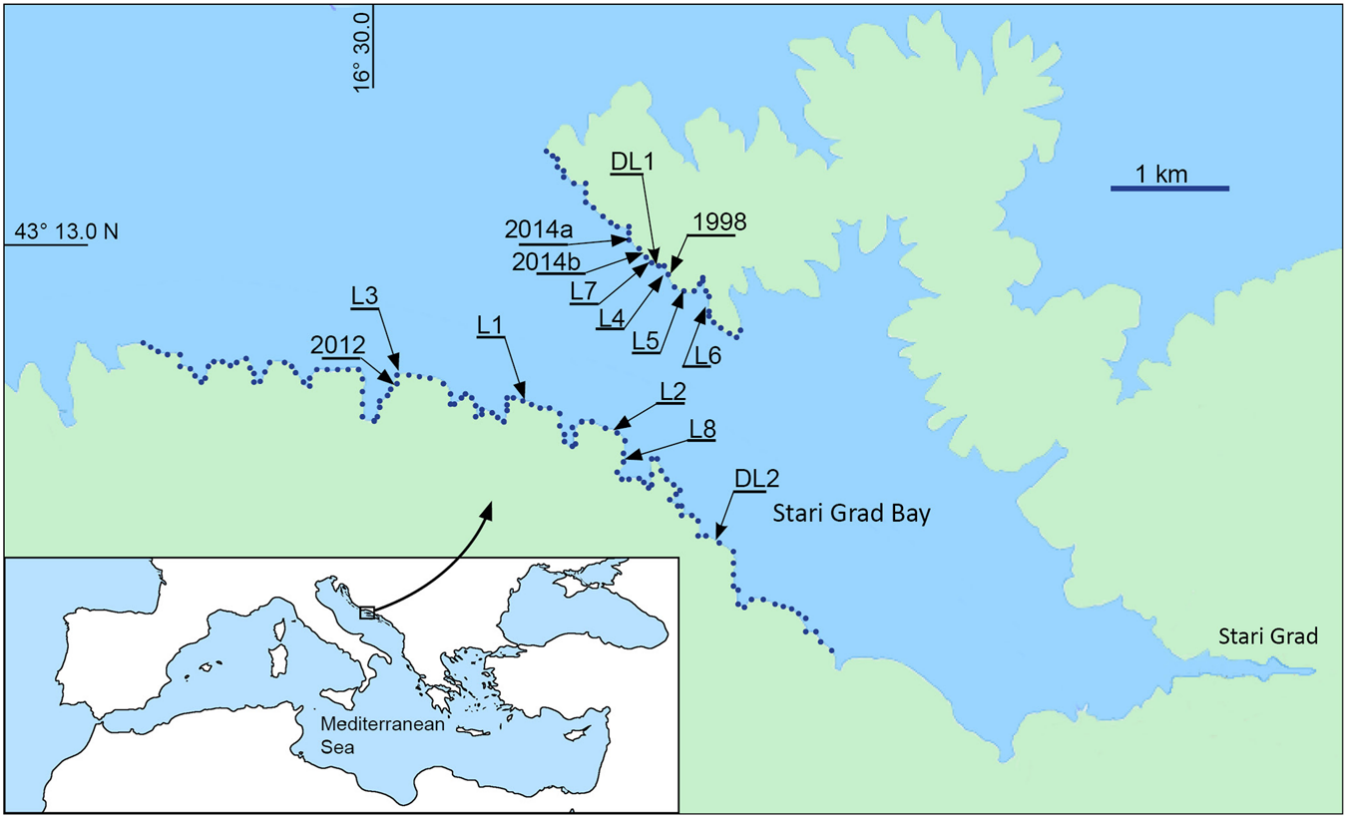
The study area where *Lithophaga lithophaga* mass spawning has been observed. Observed spawning locations include the preliminary observations made in 1998, 2012 and 2014 and the systematic observations made in 2015 (locations L1–L8). The dotted lines indicate the extent of the coastline inspected twice each day during the systematic observations made in 2015. DL: location of temperature data loggers. Maps were created using Adobe In Design CS5 and Photoshop CS5 software (www.adobe.com) and based on OpenStreetMap, © OpenStreetMap contributors (https://www.openstreetmap.org/copyright). The map tiles are licensed under CCBY-SA; the license terms can be found on the link: http://creativecommons.org/licenses/by-sa/2.0/.

In 1998 and 2012 we accidentally observed two episodes of mass spawning by *L. lithophaga*. As those observations were made on almost the same day and location (Fig. 2, Table 1), we hypothesised that mass reproduction in this species is a common reproductive strategy and is triggered by specific environmental factors rather than random isolated events.

**Table 1:** Observed spawning events by *Lithophaga lithophaga* at each of the detected locations (Fig. 2).

Location code	Initial observation			Total elapsed time (h)	Total Affected coastline (m)
	Date	Time	Affected coastline (m)		
1998*	19.08.1998	13 h	50	—	—
2012*	24.08.2012	16 h	50	—	—
2014a*	10.09.2014	12 h	30	—	—
2014b*	10.09.2014	19 h	2	—	—
L1	31.08.2015	12 h	1,5	>60	410
L2	01.09.2015	24 h	50	>60	300
L3	02.09.2015	12 h	20	>24	40
L4	02.09.2015	24 h	10	>48	100
L5	03.09.2015	24 h	15	>24	50
L6*	03.09.2015	24 h	15	>>72	>>100
L7*	04.09.2015	12 h	10	>>60	>>60
L8	04.09.2015	12 h	10	>24	10

Affected coastline on the date/time when spawning was observed initially and total elapsed time and total affected coastline at each location.

*Unknown time of spawning termination.

>Due to the adopted field observation methodology (every 12 h) total elapsed time can be a maximum of 24 h longer than the indicated value.

>>As spawning termination was not detected, total elapsed time can be significantly longer.

In 2014, during five days of field research (at a similar time of the year and location) we developed a methodology for monitoring spawning activity over a 10 km long stretch of coastline and, by applying it, observed two additional mass spawning events. Following on from those preliminary observations and using the developed methodology, in 2015 we undertook a systematic field monitoring of *L. lithophaga* spawning. The aim of this was to test our initial hypothesis that *L. lithophaga* might engage in mass spawning and, if so, gather environmental data necessary to identify the possible cue(s) that may trigger such an event(s).

Herein, we provide the first published information on co-ordinated mass spawning by *L. lithophaga*. We consider this to probably represent the greatest *in situ* spawning event ever recorded for any marine and freshwater mollusc. Based on field observations, we quantify the observed spawning process, relate it to prevailing oceanographic parameters as well as with phases of the moon and argue that it is probably co-ordinated by chemical cue(s). We will demonstrate why mass spawning should be regarded as a significant element in the overall reproductive strategy of this species.

## Results

### Preliminary observations and information from fishermen

We observed mass spawning of *Lithophaga lithophaga* accidentally during snorkelling expeditions, almost on the same day of the year, in the late summers of 1998 and 2012 (Fig. 2, Table 1). In 2014, during 5 days of intensive snorkelling in the same and nearby areas, we did not observe a spawning event. As a result of the change in observation methods (using a boat instead of snorkelling) in 2014 we were able to increase the surveillance area and identify two spawning locations on the last day of this survey period (Fig. 2, Table 1, Supplementary Videos S1–S3). The first spawning area was recorded at 12 h (affecting ca 30 m of coastline between 0 and 5 m depth) and this increased to around 50 m of coastline at 20 h on the same day. At 19 h we identified a second spawning area affecting ca 2 m of coastline (Fig. 2, Table 1). Observations then ceased due to storm conditions.

Among more than 100 questioned local sports and professional spear gun fishermen (Supplementary file), four reported observing “white clouds” over the shallow rocky bottom in the late summer, which we consider to also possibly indicate spawning by *L. lithophaga*. Our preliminary observations and the information gleaned from fishermen provided us with several important facts, which we used in the systematic observations undertaken in 2015. That is, spawning occurs at the end of summer, is not necessarily connected with a full moon, and might last for more than half a day. This could be detected from a boat by driving along the coastline at a speed of 4 km/h at 3 m from it, as follows.

### Systematic spawning observations

During 13 days of field work (from 25 August to 6 September 2015), we observed *L. lithophaga* spawning during 7 consecutive days from 31 August to 6 September, at 8 locations (Table 1, Fig. 2, Supplementary Figs S5–S12). Field work stopped on the afternoon of 6 September due to storm conditions.

**Figure 3:**
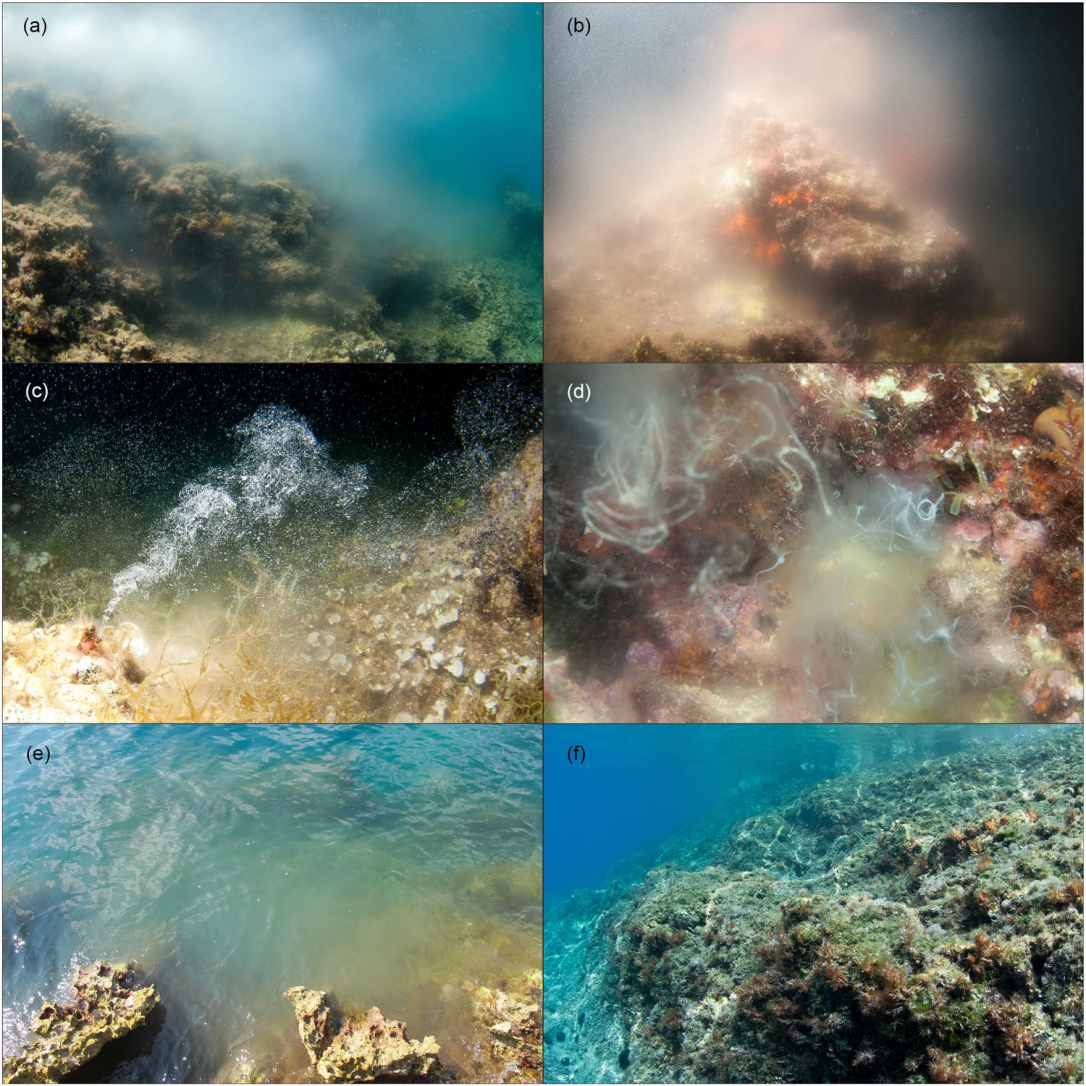
Mass synchronised spawning by *Lithophaga lithophaga*. (**a**) Margin of the spawning zone at a depth of between 0 and 5 m at mid-day. (**b**) Spawning area at around midnight and at a depth of between 1 and 3 m. (**c**) A female individual releasing two streams of eggs (24 h). (**d**) A male releasing a grey cloud of sperm (12 h). (**e**) Spawning areas can be seen readily from the boat due to the brownish-grey colour of the water. (**f**) The high transparency of the seawater close to the spawning area and typical structure of the sea bed between 0 and 5 m in the study area.

Spawning locations were identified easily from the boat as the events look like a cloud of waste water runoff. That is, the seawater becomes grey-brown and transparency falls to <0.5 m (Fig. 3e). Spawning locations have a smell of the bivalve, which on a few occasions was noticed before the spawning event was seen visually. The twice daily frequency of monitoring enabled us to detect new spawning locations in their early phases of development, within 12 h after the initial onset of such events. The smallest detected spawning location was ~1 m in diameter (depth 1.5 m) (Location 1 in Table 1, Supplementary Fig. S5). The total area of each observed spawning location extended between 40 to >400 m of coastline, affecting the sea bed at depths of between 0 and 12 m. Spawning events at a given location lasted from between 12 h to >72 h (Table 1, Supplementary Figs S5–S12).

**Figure 4:**
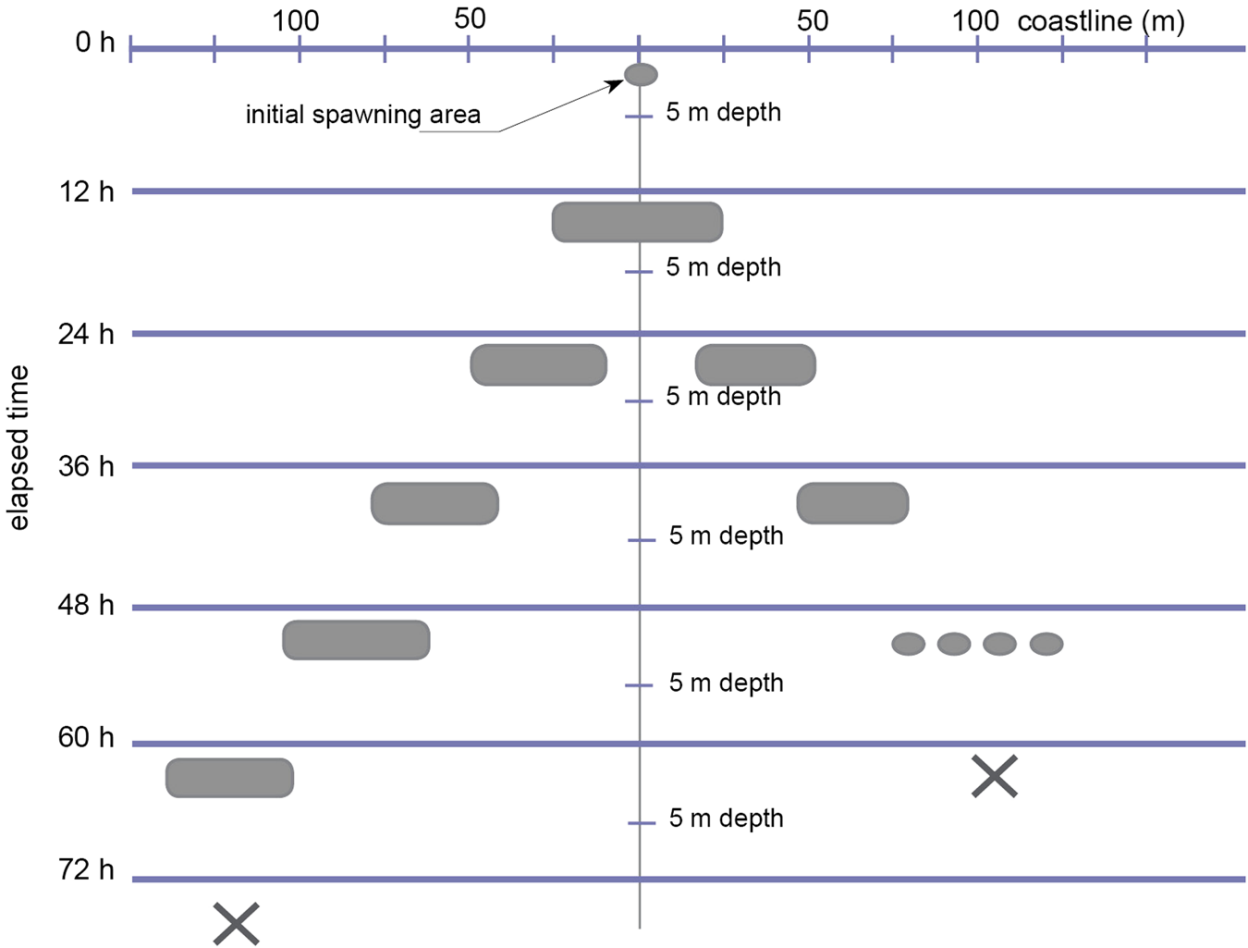
Diagrammatic illustration of the development of a *Lithophaga lithophaga* synchronised spawning area. Spawning was initiated by either one or a few individuals and progressively affected spatially close neighbours. Such a reproductive wave might finally spread over several hundred metres of coastline and last longer than three days.

The majority of the observed spawning locations showed a similar pattern of development that can be summarised as follows (Fig. 4). (i), Spawning was initiated by the release of gametes from a few neighbouring individuals, that is, within one metre of each other. (ii), Spawning affected spatially closer individuals progressively. (iii), During the first 12 h, the spawning area grew and came to affect 40–60 m of the coastline from 0 to 5 m depth. (iv), 24 h after initiation, the spawning area was expanded to cover an extra 20–30 m on each side of the coastline, while the area which had been active at t = 0–12 h, ceased gamete release. The pattern of enlargement of the spawning area, that is, expansion by an additional 20–30 m on each side of the coastline every 12 h, but then ceasing after 12 h, commonly lasted for a total of 60 h when spawning gradually ceased at the location overall (Fig. 4).

The spawning events attracted several fish species. Among these, the most obvious were golden grey mullet (*Liza aurata*) (Supplemetary Fig. S5), damselfish (*Chromis chromis*) and saddled seabream (*Oblada melanura*) (Supplementary Videos S1 and S2). No surface slicks of gametes as a result of the mass spawning events were observed.

### Gamete release

Water transparency in an active spawning area typically fell to 0 m (Fig. 3, Supplementary Information). In the waters bordering the spawning zones, an influx of clean seawater allowed observations upon and photo documentation of the events (Supplementary Videos S1 and S2). *Lithophaga lithophaga* individuals released gametes from their exhalant siphons, which projected only a few millimetres from the rock surface (Supplementary Video S3). Gametes were ejected periodically, usually at intervals of <30 seconds. Female *L. lithophaga* individuals released eggs in two parallel streams (Fig. 3c) reflecting how the paired ovaries and gonadal apertures open into their supra-branchial chambers^14^ (Fig. 4e).

Each egg is visible with the naked eye. Male *L. lithophaga* released sperm in the form of a grey cloud in which no structures were visible (Fig. 3d). In the spawning area, gamete expulsion could be initiated artificially by generating water movement close to the siphons, this resulting in the instant release of gametes. Gamete clouds slowly rose in the water column and concentrated close to the sea surface (Fig. 3a).

The numbers of reproductively active *L. lithophaga* per m^2^ of the rocky bottom were not possible to ascertain accurately due to zero visibility and periodic gamete release but we estimated there to be >50 per m^2^.

### Environmental parameters

All spawning events were observed at the end of August and beginning of September, which corresponds to the period soon after when maximum seawater temperatures are recorded for the Adriatic Sea. During systematic observations made in 2015, the weather that corresponded with the onset of observed spawning was generally stable with no clouds except on the penultimate observation day. Seawater temperature measured at two locations at 1.5 m depth during the field research was between 25.4 °C and 28.3 °C with an usual diurnal oscillation of about 1.5 °C (Supplementary Fig. S4). Wind speed was minimal, with up to Force 2 on the Beaufort Scale usually between 12 h and 18 h while during the night it was 0. Local current speeds were minimal thereby allowing the vertical lifting of gamete clouds. On the last two days of observations in 2015, the weather became stormy resulting in an end to field work. Similar calm and stable environmental conditions had also prevailed during the initial observation periods in 1998, 2012 and 2014.

**Figure 5:**
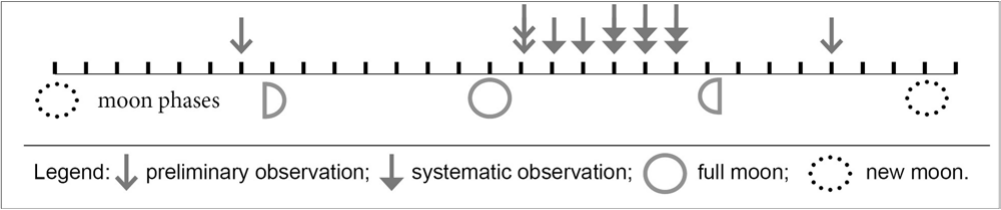
Observed synchronised spawning events by *Lithophaga lithophaga* in relation to the phases of the moon. Each arrow indicates when a spawning event at a given location was observed initially (Fig. 2). The exact observation times are identified in Table 1.

The onset of spawning by *L. lithophaga* showed a generalised correlation with the lunar phases as maximal events occurred over a six day period after the full moon (Fig. 5).

## Discussion

*In situ* observations of spawning behaviour among marine invertebrates, aside from corals, have been documented for small number of species^15–18^. The reproduction events of tropical hermatypic corals are well-studied globally^19–22^. Mass spawning by *Lithophaga lithophaga*, observed *in situ* and described herein for the first time, constitutes an additional example of synchronised reproductive activity by a marine invertebrate. Further, *in situ* observed spawning events of such synchronicity and scale have not previously been reported upon for any other marine or freshwater mollusc.

Co-ordinated gamete release to effect fertilization in sessile marine invertebrates is a crucial element of their overall reproductive traits. Within these, there are two important strategies the adoption of which can increase reproductive success: (i), the synchronous release of gametes and (ii), spawning under favourable environmental conditions. A third strategy, the aggregation of fertile individuals, is common in mobile benthic invertebrates as well as in other vagile marine species exhibiting external fertilization^23^. Synchronised reproduction in the majority of marine invertebrates (including the broadcast spawning species of hermatypic corals) is thought to be orchestrated by combinations of different environmental factors such as the phases of the moon, tidal cycles (also related to the moon’s cycle), time of day, food supply, and seawater temperature. These serve as proximate cues to initiate development and gamete release^15–17,21,23,24^. It seems, however, that none of these environmental cues, except for phases of the moon, is especially important and adopted in relation to mass spawning by *L. lithophaga*.

As has been suggested by gonadal index studies and, herein, confirmed by our field observations, *L. lithophaga* spawns at the end of the northern hemisphere summer, either during the period of or soon after the highest annual water temperatures^7,12,13^. It has been proposed that periods of spawning coincide also with changes in salinity and dissolved oxygen^7,12^. These conclusions were, however, based on monthly obtained data of gonadal index and abiotic factors and such information can, therefore, only be an indicator of prevailing sea characteristics and not, necessarily, spawning cues.

Rapid adjusted temperature change is a basic laboratory method used to initiate gamete release in bivalve molluscs^25^, including the eastern oyster *Crassostrea virginica*^26^. No significant overall variations in seawater temperature and only normal, slight, diurnal changes in such a potential cue were, however, detected during our observations of *L. lithophaga* spawning (Supplementary Fig. S4).

It is widely recognised that the presence of planktonic algal ectocrines is important in the induction of spawning in a variety of bivalves including *Crassostrea virginica*^27^ and *Mytilus edulis*^28^. No evidence was obtained in our study, however, to indicate the presence of any phytoplankton blooms at the times of witnessed spawning events by *L. lithophaga*. The onset of gamete release in *L. lithophaga* was, moreover, in all recorded situations, connected with a prolonged period of calm weather and, therefore, no abrupt changes in salinity, dissolved oxygen or food availability could be identified as potential exogenous factors triggering spawning. We do, however, speculate that a calm sea is an important pre-requisite for the development of synchronised spawning events (see below). As tidal oscillations in the study area were <0.4 m, it is also unlikely that they have any influence on spawning initiation.

The lunar cycle, which has been demonstrated to have an important role in mass spawning in several sessile marine organisms^29^, with a co-ordinating influence at a greater spatial scale, might be involved in the induction of spawning by *L. lithophaga*. The majority of spawning events by *L. lithophaga* occurred during the period between full moon and its last quarter. Such a strict connection was, however, disturbed by two observed spawning events, one before the first quarter and a second before the new moon (Fig. 5). In tropical species *of Lithophaga*, that is, *L. lessepsiana*, *L. simplex* and *L. purpurea*, although rare and observed under laboratory conditions, gamete release occurred primarily during the last quarter and the new moon^30^. This is not, however, the case for *L. lithophaga*. Additionally, the peak in spawning activity exhibited by *L. lithophaga* identified in this study and which coincided with the period after the full moon, might be a detection bias related to relatively limited field observations.

The onset of spawning by *L. lithophaga* is not considered to be related to the period of the day as in many sessile marine broadcast spawners which mostly spawn at night^24^. Night spawning is an evolutionary adaptation to avoid predation as a majority of nektonic plankton feeders (mainly fishes) are diurnal species. As spawning in *L. lithophaga* in some locations lasts for a few days (Table 1), the release of gametes in connection with a definable period of the day is obviously not a mechanism for predation reduction.

Unlike broadcast spawners such as corals, spawning in *L. lithophaga* does not occur synchronously over a geographically large area. Instead, simultaneous spawning affects closely adjacent individuals over smaller areas of tens of metres of coastline. Spawning in *L. lithophaga* commences with a few individuals and then spreads slowly throughout neighbouring, conspecific, individuals along between 15–30 m of coastline per 12 h (Fig. 4). A similar situation has been observed in the sea urchin *Evechinus chloroticus*, where large spawning events are often triggered by those of an initially small number of conspecifics within a population^23^. As in *E. chloroticus* and other benthic invertebrates which have unpredictable spawning times (unlike the predictability of corals), the presence of pheromones has been proposed as a spawning stimulus^31^. Some bivalves can, moreover, under laboratory conditions, be stimulated to release gametes by adding either sperm or pheromones to their seawater^32^. Although the addition of sperm as a laboratory technique has been used unsuccessfully to induce spawning in several tropical *Lithophaga* species^33^, the ‘gamete to gamete’ induction of spawning seems to be the most likely proximate cue in synchronising gamete release in *L. lithophaga*. The threshold concentration of reproductive cells (or associated chemicals like pheromones) in the seawater, when reached, stimulates neighbouring individuals to start releasing their gametes which results in a reproductive wave along the shoreline (Fig. 4).

It could be argued, however, that if either a chemical or ‘gamete to gamete’ cue was present, an extensive gamete cloud should initiate spawning by *L. lithophaga* over a spatially broader area and occur within a relatively short time. To the contrary, our observations suggest that the gamete cloud lifts in the water column and probably does not, therefore, transmit a localised cue far from the actively spawning group of conspecific individuals. The threshold concentration of gametes in the water must be obviously high in order to trigger neighbouring individuals to discharge their gametes. Such a high threshold concentration that initiates spawning in localised conspecifics is unlikely to prevail in the situation of strong hydrodynamics. Calm weather and, therefore, a calm sea and minimal currents (situations in which we observed *L. lithophaga* to be spawning) seem to be a prerequisite for and play a crucial role in the mass spawning events exhibited by this species.

‘Gamete to gamete’ stimulation in the case of *L. lithophaga* spawning has the potential to be demonstrated in aquaria and under experimental conditions. It has, however, proven difficult to induce spawning by other lithophagine species in the laboratory^30^, and some classical aquaculture methods for the induction of gamete release in other bivalves (increases and decreases in temperature, injection of KCl, rapid shaking), when applied to different tropical species of *Lithophaga* as well as *L. lithophaga*, did not obtain the expected results^33,34^.

In conclusion, according to the herein described field observations and earlier studies of reproduction, mass spawning events in *L. lithophaga* occur in the following sequence: (1), maximal annual water temperatures influence the maturation of the gonads; (2), a calm sea state and minimal currents provide the environmental prerequisites necessary for a mass, synchronised, spawning event to occur; (3), gamete release by a few individuals triggers the mass spawning event; (4) as a result of ‘gamete to gamete’ induction the spawning area spreads like a reproductive wave and expands by 20–30 m every 12 h; (5) spawning finally might affect >400 m of rocky coastline and last >74 h at any given location.

There is one another question that arises from this field study of *L. lithophaga* spawning. How is it possible that such a large, long-lasting and noticeable event produced by one of the most common and, in many respects, one of the most important Mediterranean species, has remained undescribed? The answer is simple: underwater observations and research have been limited hitherto. Our results highlight the importance of such basic marine biological studies. Mass spawning by *L. lithophaga* is as visually impressive as the mass spawning events described for tropical corals and is proof that there are still fascinating biological events to be discovered, even in our own backyard. Just as the public’s awareness of the threats to coral reefs has benefited from observations on mass spawning events, so that of *L. lithophaga* might be similarly useful in raising awareness of the shallow water Mediterranean biocenoses and of the importance of regional marine conservation. We might expect that due to legal protection of the *L. lithophaga* and therefore slow but progressive population restoration, formation and observation of the mass spawning events will occur more often.

## Materials and Methods

### The study area

Observations of *Lithophaga lithophaga* spawning were undertaken in Stari Grad Bay, Island of Hvar (Adriatic Sea, Croatia) (43.20°N–16.53°E) (Fig. 2). The shallow sea bed of the bay is rocky and comprises compacted limestone with a typical karst morphology and high rugosity (see Supplementary Information). Bottom inclination between depths of 0 and 5 m varies from sub-vertical, vertical, inclined to horizontal but, overall, is ~45°. *Lithophaga lithophaga* is probably present continuously throughout the coastline with the highest densities occurring at between 0 and 5 m depth. The majority of this rocky bottom has over the last decades been damaged by the harvesting of *L. lithophaga*, especially intensively in the 1980s (unpublished data).

### Preliminary observation

Initial observations of *L. lithophaga* spawning in Stari Grad Bay occurred accidentally in 1998 and 2012, at the end of the respective summers (Fig. 2, Table 1). In 2014, five days of field observations were made within the same area and during the same period of the year as in 1998 and 2012 with the aim of recording of *L. lithophaga* spawning. Intensive snorkelling activities during different times of the day were undertaken at several locations throughout Stari Grad Bay. On the 5^th^ day, observations were performed from a rigid-hulled inflatable boat (3.8 m, 18 hp engine), by driving slowly close to the coastline (see below). With such a method, we increased the extent of coastline under observation considerably and this resulted in the recording of two spawning events (Locations 2014a, b in Fig. 2). Field observations were, unfortunately, then terminated due to storms. All these preliminary observations have, however, provided the essential input necessary to organise the more systematic field research on *L. lithophaga* spawning conducted in 2015.

Since our first accidental observation of *L. lithophaga* spawning in 1998, we have also interviewed local water sports enthusiasts and professional spear gun fishermen about observations of “white clouds near the shallow rocky sea bed” (Supplementary information).

### Systematic spawning observation

Field observations were made from 25 August until 6 September 2015 in Stari Grad Bay (Fig. 2). During this period, we searched for *L. lithophaga* spawning activities by observations from the rigid-hulled inflatable boat described above. Approximately 13 km of rocky coastline were inspected twice each day, between 10–14 h and 22–02 h (Fig. 2). Night observations were made using a hand torch. The boat was driven at a speed of ~4 km/h at a distance of ~3 m from the coastline. Water transparency allowed observations of the sea bed from the boat to a depth of 5 m.

In the situation of uncertain observations, confirmatory (or not), inspections were undertaken by snorkelling. Sea state during the field observations was mostly calm to maximum sea state 2, according to the Douglas Sea Scale.

Each detected spawning area was also surveyed by snorkelling and SCUBA diving, two or three times each day (morning, afternoon and night) with the aim of obtaining underwater photographic and video documentation. This resulted in the drawing up of a precise map of the spawning area and records of associated biological features. Seawater temperature was measured over the entire period of field observations at 10 minute intervals using data loggers (HOBO Pendant UA-002–64) set at a depth of 1.5 m at two locations (Fig. 2).

## Electronic supplementary material

Video S1

Video S2

Video S3

Supplementary Information
