# Supplementary material for: Mass spawning by the date mussel Lithophaga lithophaga
Source: Sci Rep. 2018 Jul 17;8:10781. doi: 10.1038/s41598-018-28826-8 (PMC6050253; doi:10.1038/s41598-018-28826-8)
Supplement: Supplementary file 4 — Supplementary Information [file 41598_2018_28826_MOESM4_ESM.pdf]

Supplementary information

Mass spawning by the date mussel *Lithophaga lithophaga*

A. Žuljević, M. Despalatović, I. Cvitković, B. Morton & B. Antolić

Supplementary Videos

Video S1. Spawning area of *Lithophaga lithophaga* detected in 2014 (location code 2014a in Figure 2). Transect throughout spawning zone parallel to the coast and different aspect of this massive reproduction event.

Video S2. Different views in the spawning zone of *Lithophaga lithophaga* detected in 2014 (location code 2014a in Figure 2).

Video S3. Gamete release by male and female individuals of *Lithophaga lithophaga* during massive spawning detected in 2014 (location code 2014a in Figure 2).

Supplementary Figures

Figure S4. Seawater temperature at two locations during field research in 2015 (location codes DL1 and DL2 in Figure 2). Seawater temperature was measured at 10 minute intervals using data loggers (HOBO Pendant UA-002-64) set at a depth of 1.5 m.

Figures S5 – S12. Additional data on *Lithophaga lithophaga* spawning observed in Stari Grad Bay (Croatia) on eight different locations during field research in 2015. Maps were created using Adobe In Design CS5 and Photoshop CS5 software (www.adobe.com) and based on OpenStreetMap, © OpenStreetMap contributors (https://www.openstreetmap.org/copyright). The map tiles are licensed under CCBY-SA; the license terms can be found on the link: http://creativecommons.org/licenses/by-sa/2.0/.

Supplementary methodology

Figure S4.

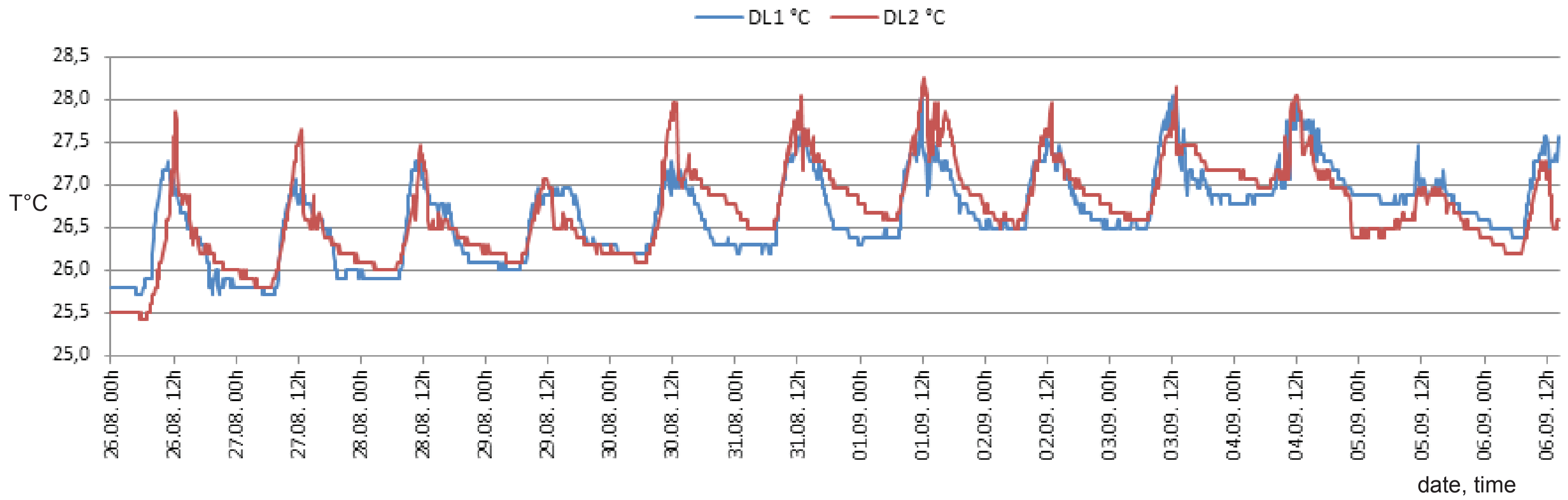
DL1 °C
DL2 °C
T°C
28,5
28,0
27,5
27,0
26,5
26,0
25,5
25,0
26.08. 00h
26.08. 12h
27.08. 00h
27.08. 12h
28.08. 00h
28.08. 12h
29.08. 00h
29.08. 12h
30.08. 00h
30.08. 12h
31.08. 00h
31.08. 12h
01.09. 00h
01.09. 12h
02.09. 00h
02.09. 12h
03.09. 00h
03.09. 12h
04.09. 00h
04.09. 12h
05.09. 00h
05.09. 12h
06.09. 00h
06.09. 12h
date, time

Figure S5. Location 1.

Coordinates: 43° 12.314’N - 16° 31.060’E
Time of first observation: 31.08.2015., 12 h
Time of last observation: 02.09.2015., 24 h
Total affected coastline: >410 m
Total elapsed time: >60 h
Depth range: 0 – 7 m

Study area:

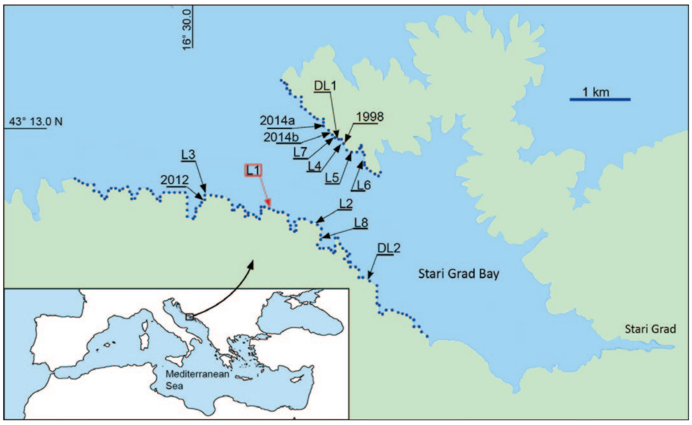

Development of spawning area:

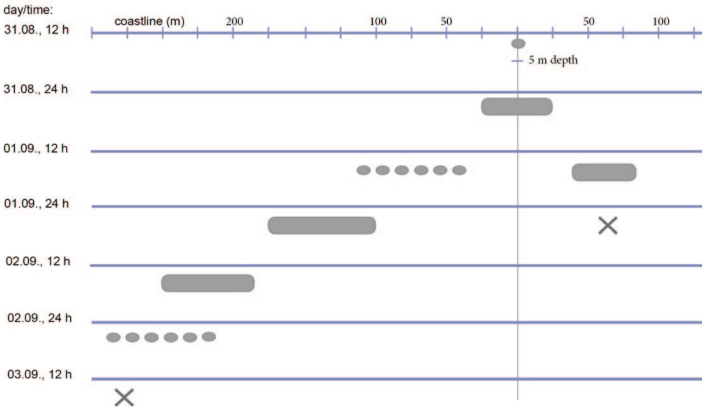

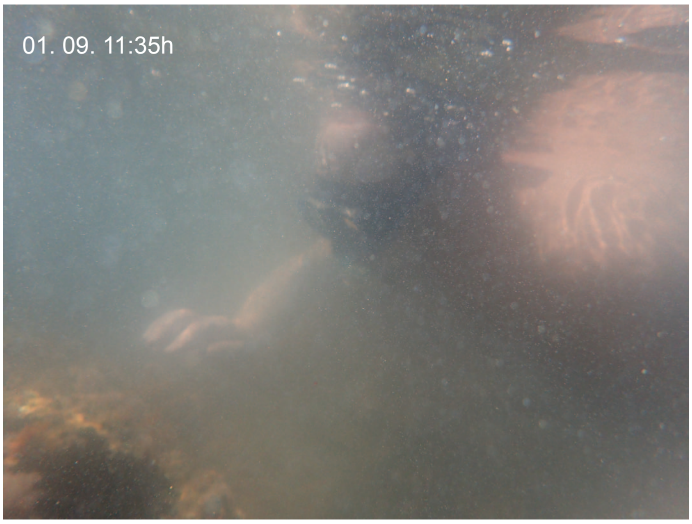

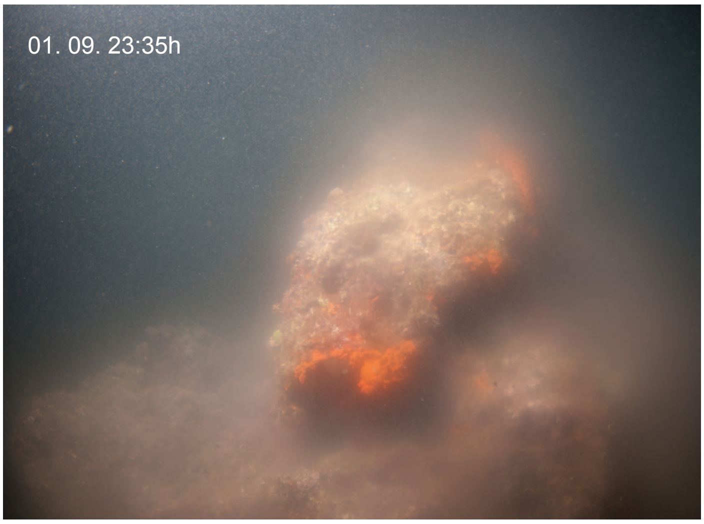

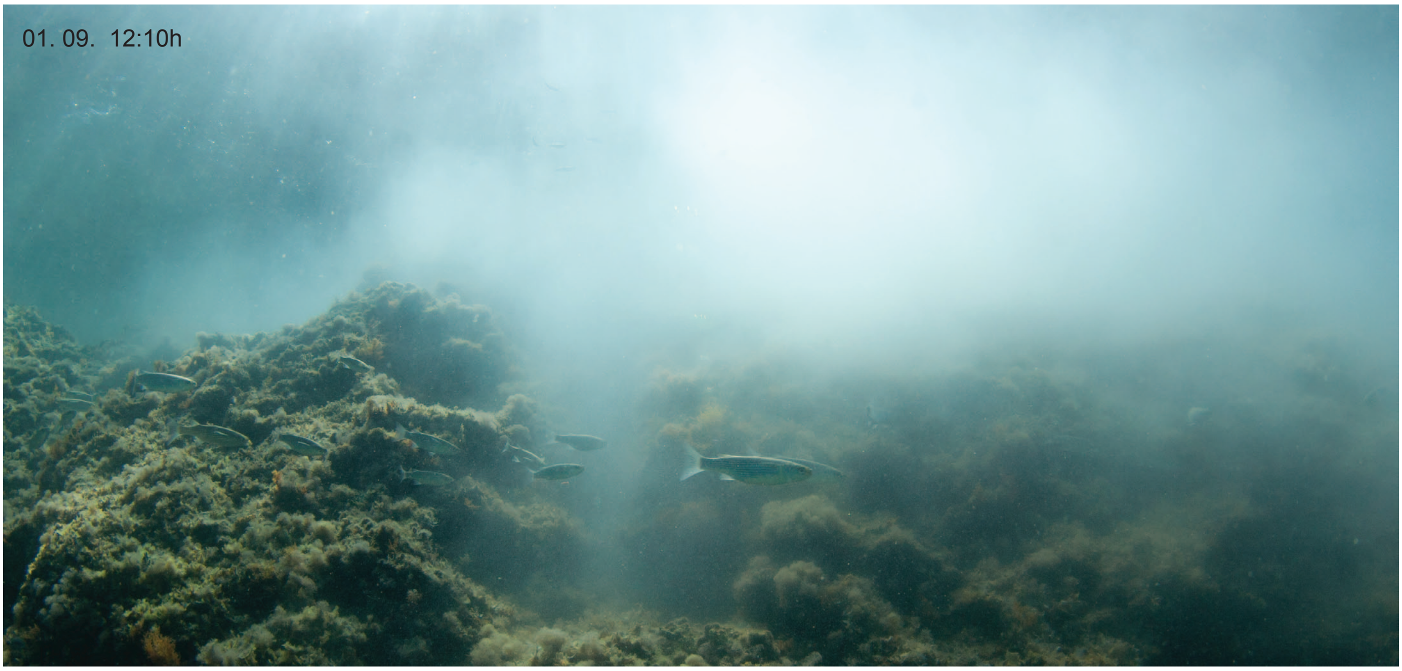

Figure S6. Location 2.

Coordinates: 43° 12.128’N - 16° 31.605’E
Time of first observation: 01.09.2015., 24 h
Time of last observation: 04.09.2015., 12 h
Total affected coastline: >300 m
Total elapsed time: >60 h
Depth range: 0 – 7 m

Study area:

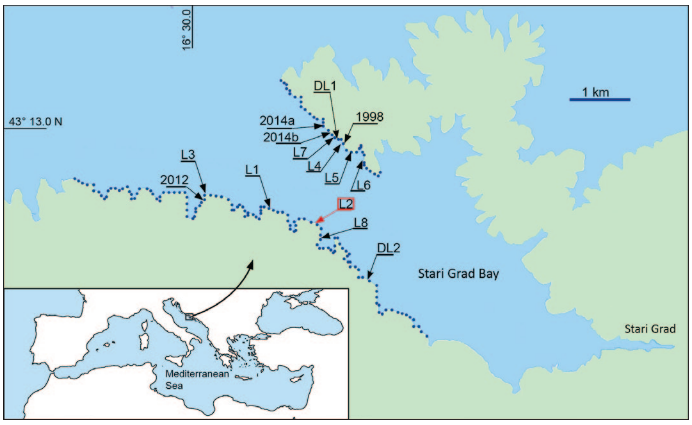

Development of spawning area:

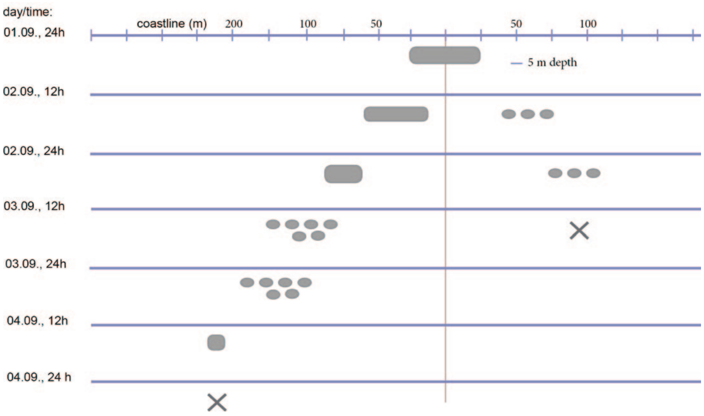

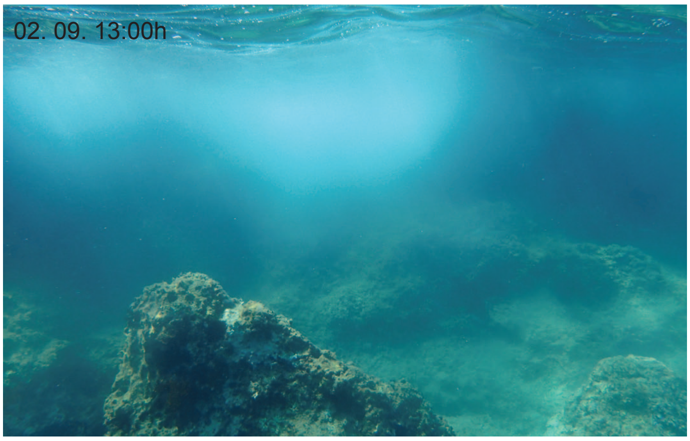

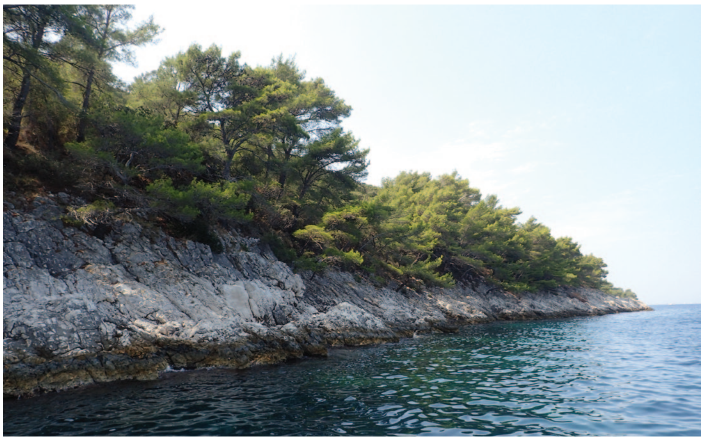

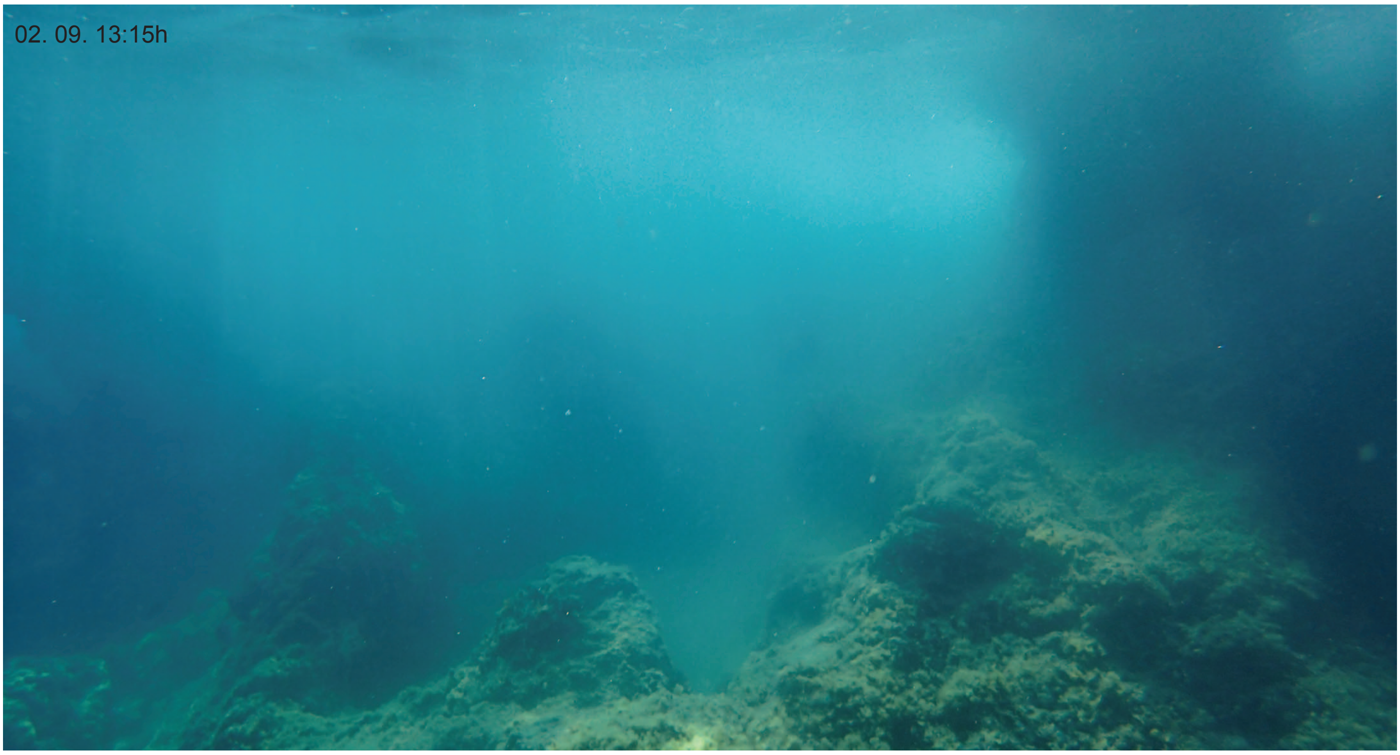

Figure S7. Location 3.

Coordinates:	43° 12. 455'N - 16° 30.215'E
Time of first observation:	02.09.2015., 12 h
Time of last observation:	02.09.2015., 24 h
Total affected coastline:	>40 m
Total elapsed time:	>24 h
Depth range:	0 – 5 m

Study area:

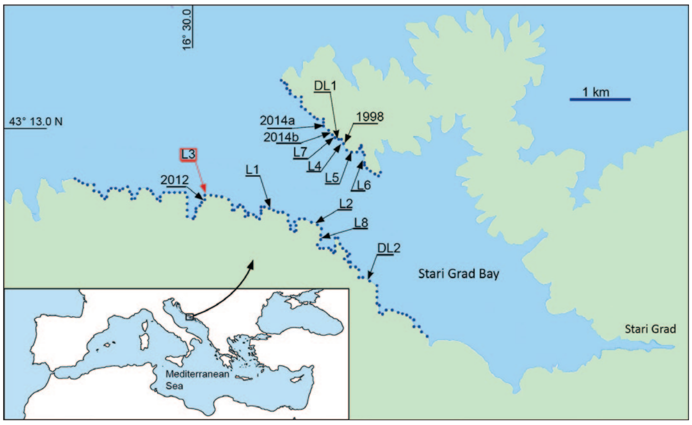

Development of spawning area:

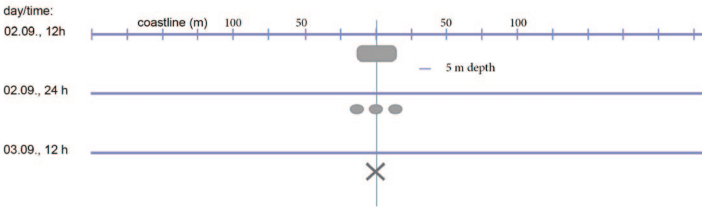

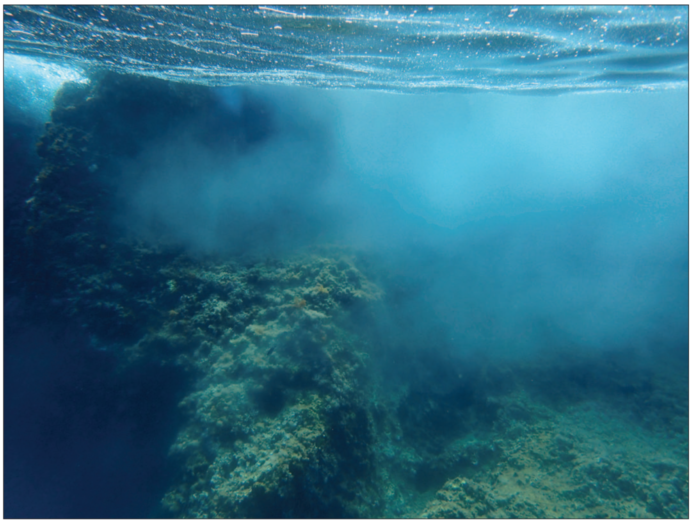

02. 09. 12:05h

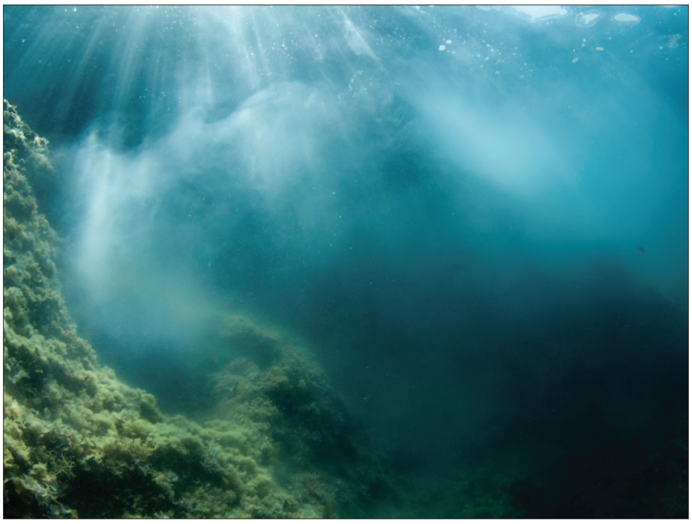

02. 09. 12:10h

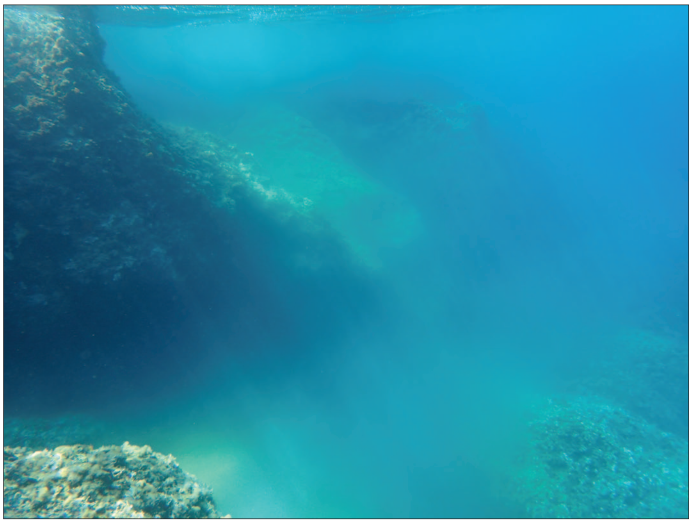

02. 09. 12:14h

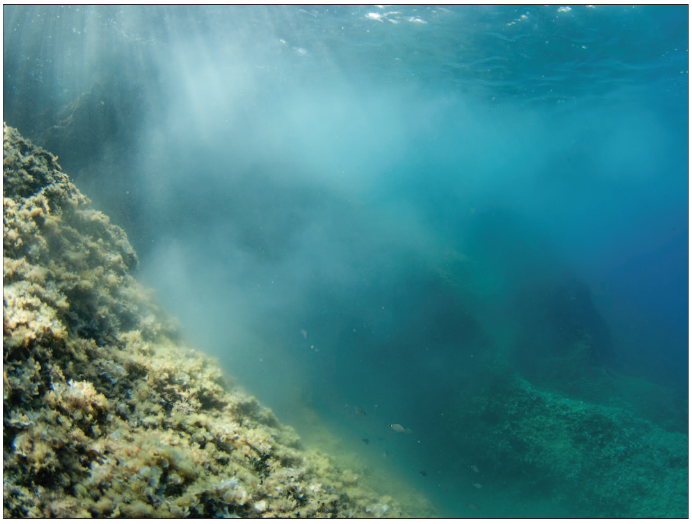

02. 09. 12:20h

Figure S8. Location 4.

Coordinates:	43° 12. 925'N - 16° 31.912'E
Time of first observation:	02.09.2015., 24 h
Time of last observation:	03.09.2015., 24 h
Total affected coastline:	>100 m
Total elapsed time:	>36 h
Depth range:	5 – 12 m

Study area:

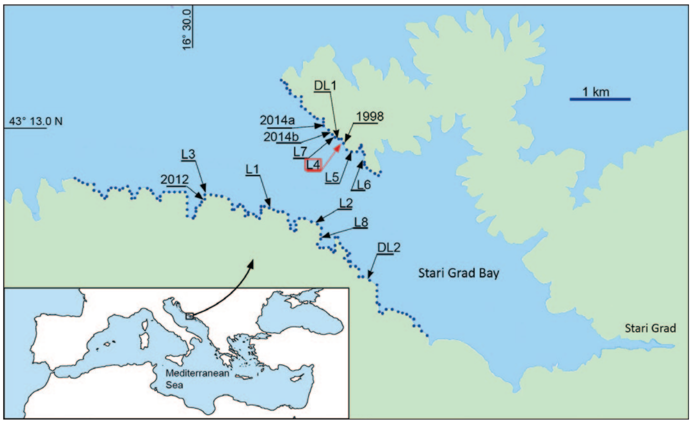

Development of spawning area:

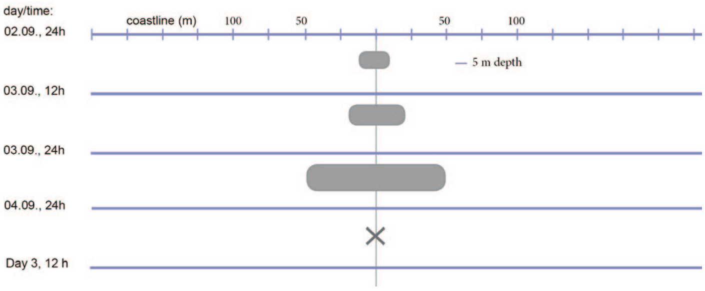

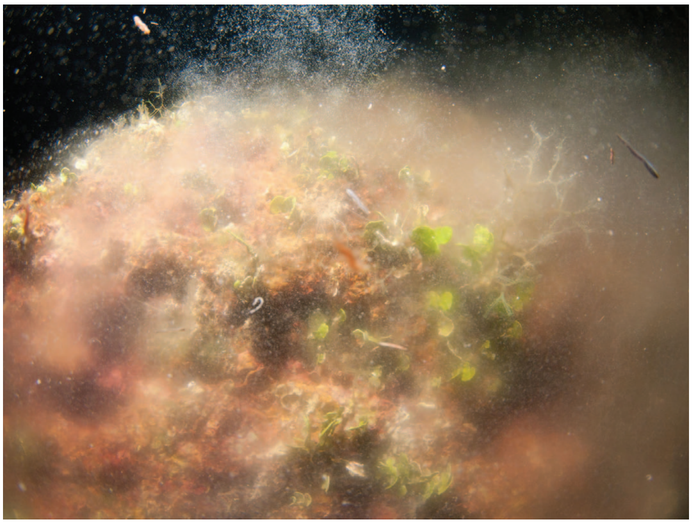

03. 09. 21:30h

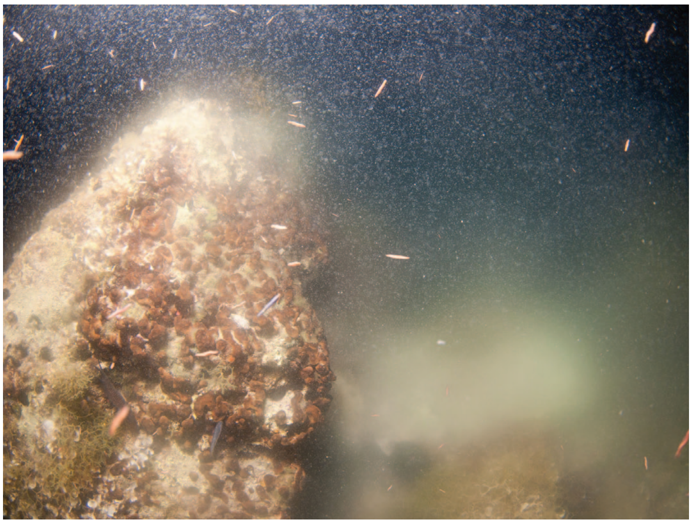

03. 09. 21:35h

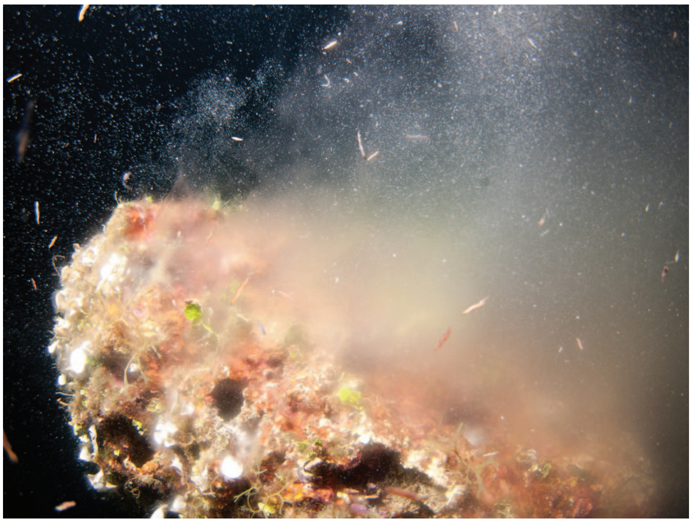

03. 09. 21:40h

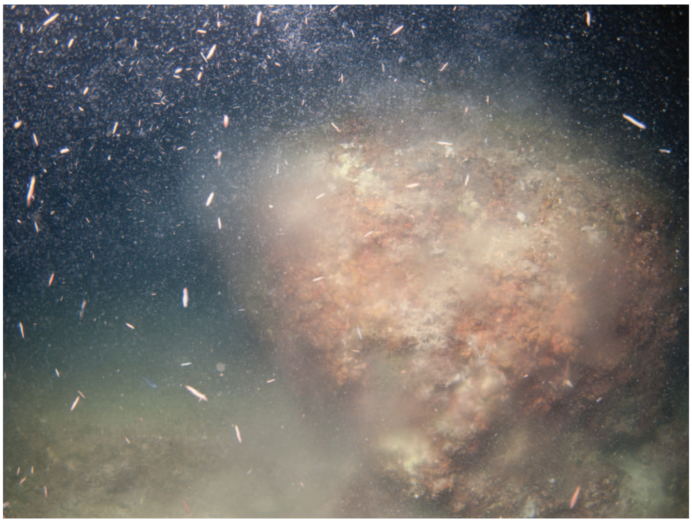

03. 09. 21:45h

Figure S9. Location 5.

Coordinates: 43° 12. 831’N - 16° 32.007’E
Time of first observation: 03.09.2015., 24 h
Time of last observation: 04.09.2015., 12 h
Total affected coastline: >75 m
Total elapsed time: >36 h
Depth range: 1 – 10 m

Study area:

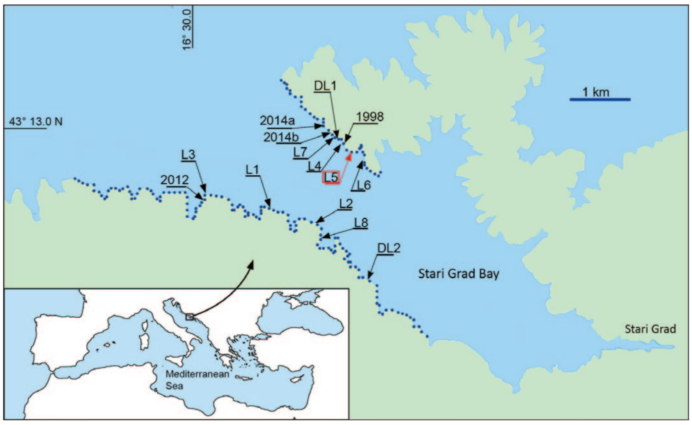

Development of spawning area:

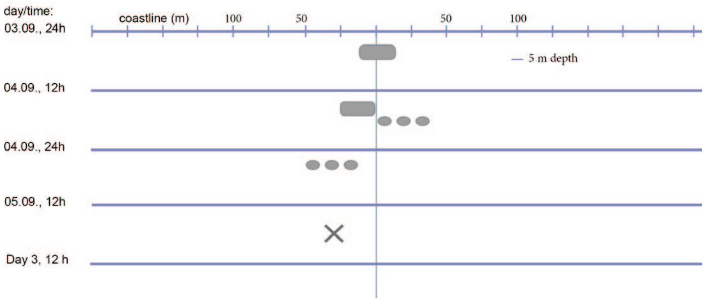

Figure S10. Location 8.

Coordinates: 43° 12. 043’N - 16° 31.660’E
Time of first observation: 04.09.2015., 12 h
Time of last observation: 04.09.2015., 24 h
Total affected coastline: >20 m
Total elapsed time: >24 h
Depth range: 3 – 7 m

Study area:

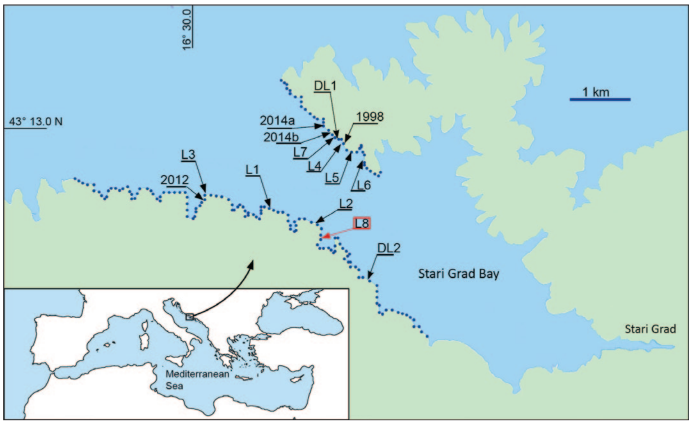

Development of spawning area:

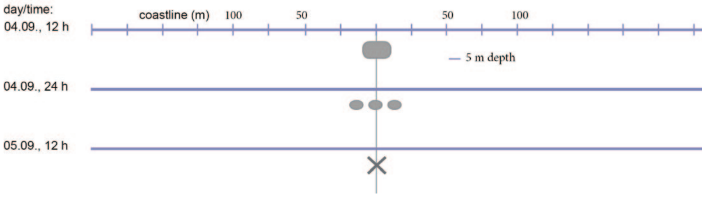

Figure S11. Location 6.

Coordinates: 43° 12. 770’N - 16° 32.192’E
Time of first observation: 03.09.2015., 24 h
Time of last observation: 06.09.2015., 12 h
Total affected coastline: >195 m
Total elapsed time: >62 h
Depth range: 1 – 7 m

Study area:

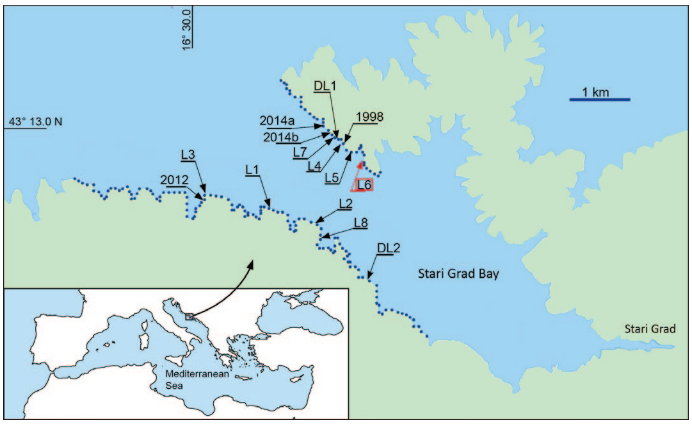

Development of spawning area:

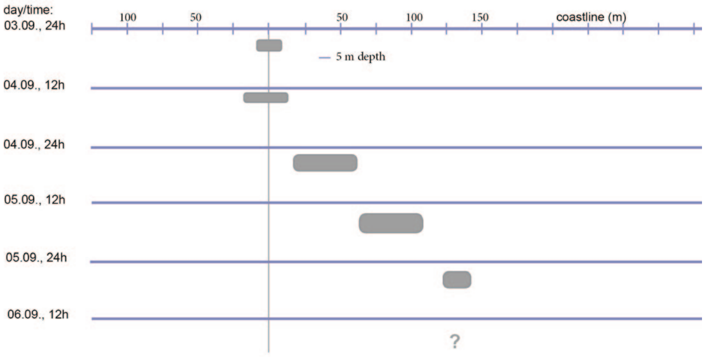

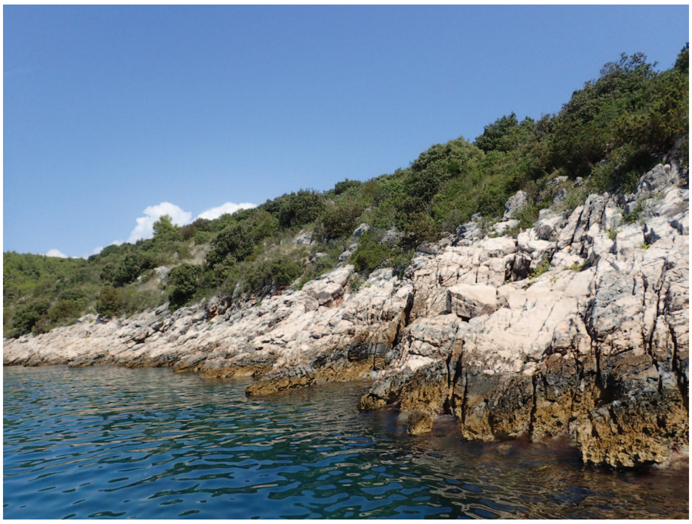

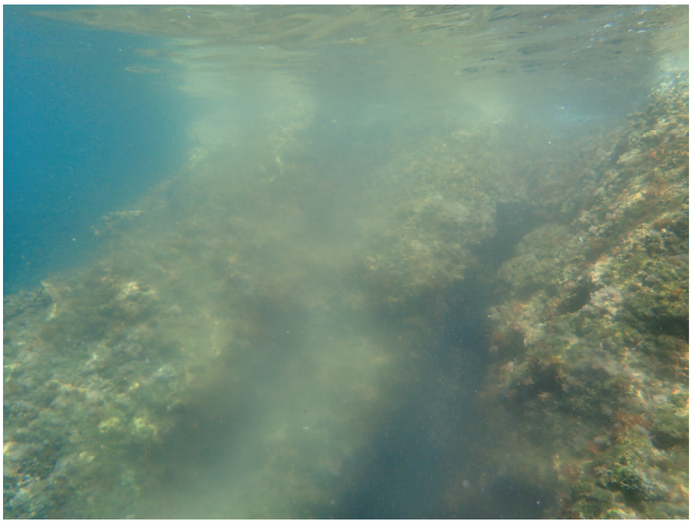

04. 09. 12:40h

Figure S12. Location 7.

Coordinates: 43° 12. 953’N - 16° 31.854’E
Time of first observation: 04.09.2015., 12 h
Time of last observation: 06.09.2015., 12 h
Total affected coastline: >100 m
Total elapsed time: >62 h
Depth range: 1 – 5 m

Study area:

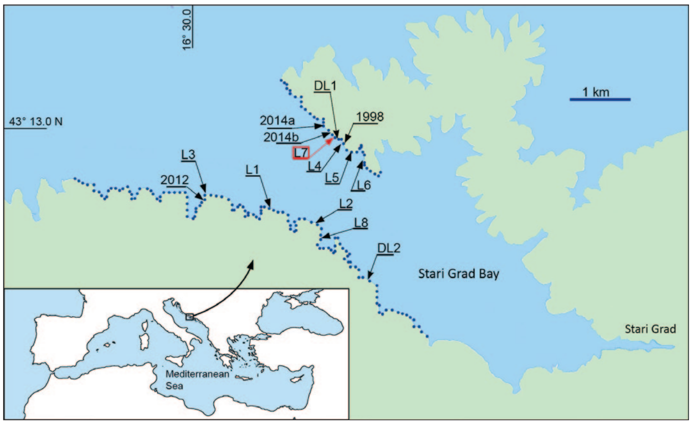

Development of spawning area:

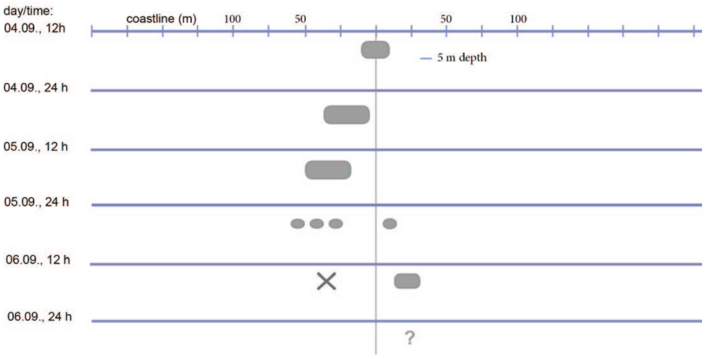

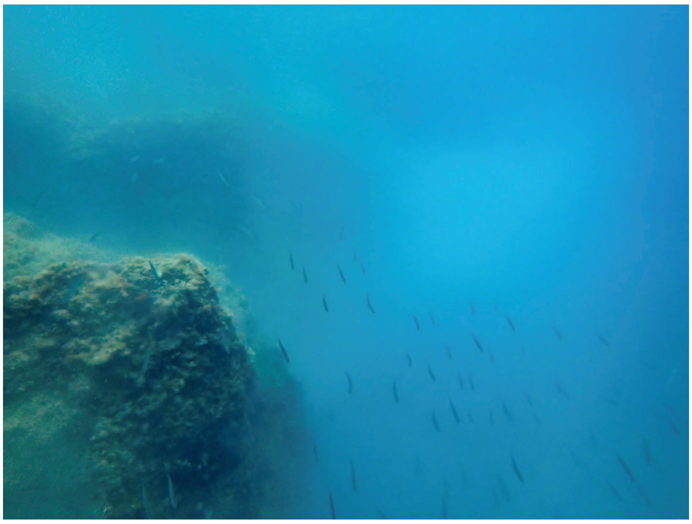

05. 09. 15:00h

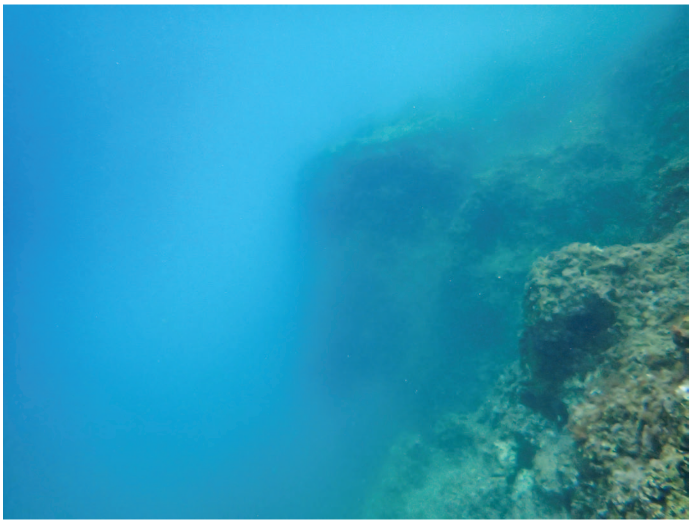

05. 09. 15:00h

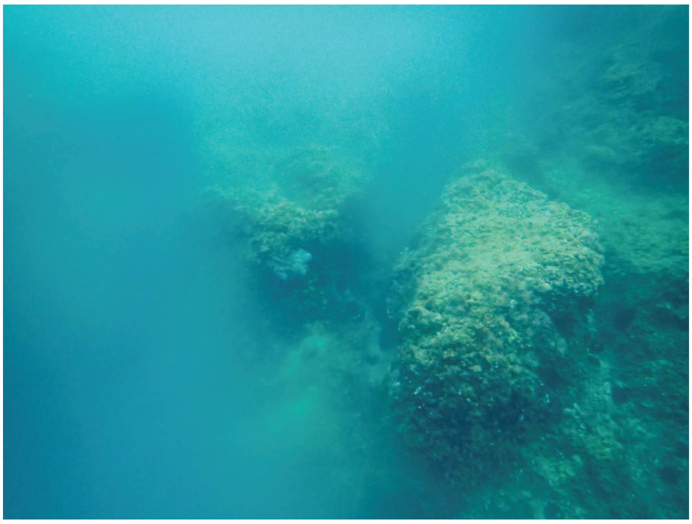

05. 09. 15:05h

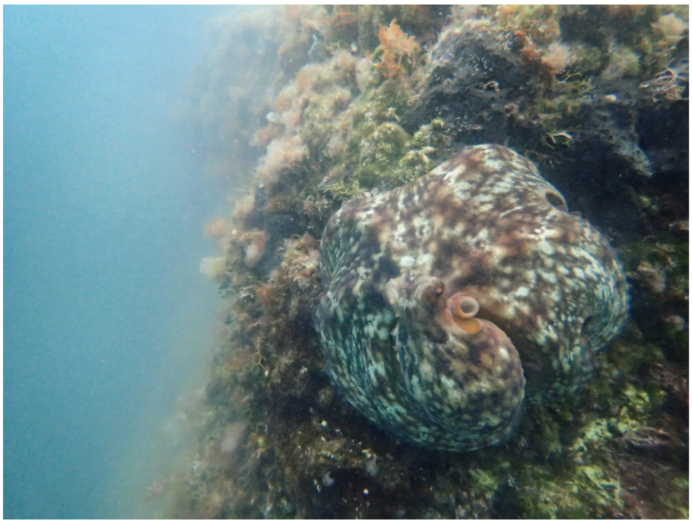

05. 09. 15:10h

Supplementary methodology – collecting of local ecological knowledge

With the aim of obtaining local ecological knowledge[1, 2] about *Lithophaga lithophaga* spawning, local water sports enthusiasts, professional spear gun fishermen and professional scuba divers were interviewed about their observations of such synchronised events, if any. We gathered information during personal interviews, during public lectures and marine biology classes in the school for professional divers.

In personal interviews, we described spawning events as "white clouds near the shallow rocky sea bed". During public lectures and school classes, we additionally presented photos and videos and asked about the similar experiences of others. We estimate that we presented the event to more than 100 persons. In any case of positive feedback, we discussed such observation in person-to-person interviews in order to determine if such observations could be correlated with a mass spawning event by *L. lithophaga* and, if so, to obtain additional data such as: date, time, location, depth, size and duration of the event, sea state and weather conditions.

References

1 Huntington HP (2000) Using traditional ecological knowledge in science: methods and applications. Ecol Appl 10: 1270–1274.

2 Anadón JD, Giménez A, Ballestar R, Pérez I (2009) Evaluation of local ecological knowledge as a method for collecting extensive data on animal abundance. Conserv Biol 23: 617–625.
